# Identification of a novel tumour microenvironment‐based prognostic biomarker in skin cutaneous melanoma

**DOI:** 10.1111/jcmm.17021

**Published:** 2021-11-10

**Authors:** Rong‐Hua Yang, Bo Liang, Jie‐Hua Li, Xiao‐Bing Pi, Kai Yu, Shi‐Jian Xiang, Ning Gu, Xiao‐Dong Chen, Si‐Tong Zhou

**Affiliations:** ^1^ Department of Burn Surgery and Skin Regeneration The First People’s Hospital of Foshan Foshan China; ^2^ Nanjing University of Chinese Medicine Nanjing China; ^3^ Department of Dermatology The First People’s Hospital of Foshan Foshan China; ^4^ Department of Emergency The Sun Yat‐sen Memorial Hospital of Sun Yat‐sen University Guangzhou China; ^5^ Department of Pharmacy Seventh Affiliated Hospital of Sun Yat‐sen University Shenzhen China; ^6^ Nanjing Hospital of Chinese Medicine Affiliated to Nanjing University of Chinese Medicine Nanjing China

**Keywords:** ESTIMATE, LASSO, prognostic biomarker, PRSS35, skin cutaneous melanoma, tumour microenvironment

## Abstract

Skin cutaneous melanoma (SKCM) is one of the most destructive skin malignancies and has attracted worldwide attention. However, there is a lack of prognostic biomarkers, especially tumour microenvironment (TME)‐based prognostic biomarkers. Therefore, there is an urgent need to investigate the TME in SKCM, as well as to identify efficient biomarkers for the diagnosis and treatment of SKCM patients. A comprehensive analysis was performed using SKCM samples from The Cancer Genome Atlas and normal samples from Genotype‐Tissue Expression. TME scores were calculated using the ESTIMATE algorithm, and differential TME scores and differentially expressed prognostic genes were successively identified. We further identified more reliable prognostic genes via least absolute shrinkage and selection operator regression analysis and constructed a prognostic prediction model to predict overall survival. Receiver operating characteristic analysis was used to evaluate the diagnostic efficacy, and Cox regression analysis was applied to explore the relationship with clinicopathological characteristics. Finally, we identified a novel prognostic biomarker and conducted a functional enrichment analysis. After considering ESTIMATEScore and tumour purity as differential TME scores, we identified 34 differentially expressed prognostic genes. Using least absolute shrinkage and selection operator regression, we identified seven potential prognostic biomarkers (SLC13A5, RBM24, IGHV3OR16‐15, PRSS35, SLC7A10, IGHV1‐69D and IGHV2‐26). Combined with receiver operating characteristic and regression analyses, we determined PRSS35 as a novel TME‐based prognostic biomarker in SKCM, and functional analysis enriched immune‐related cells, functions and signalling pathways. Our study indicated that PRSS35 could act as a potential prognostic biomarker in SKCM by investigating the TME, so as to provide new ideas and insights for the clinical diagnosis and treatment of SKCM.

## 1 BACKGROUND

Skin cutaneous melanoma (SKCM) is a malignant transformation of melanocytes derived from neural crest stem cells.^1^ Although SKCM accounts for only approximately 5% of all skin tumours, it causes more than 75% of deaths from skin tumours.^2^ Recently, the approval of targeted‐ and immune‐based therapeutic agents with substantial activity has brought new hope for the treatment of SKCM.^3^, ^4^, ^5^ However, approximately half of the patients treated with these therapies have no sustained response and display no satisfactory improvement in prognosis.^6^, ^7^ Moreover, some patients have immune‐related adverse events.^8^, ^9^, ^10^ Therefore, it is urgent and important to explore biomarkers that can identify the population that is more likely to benefit from these treatments.

Tumour cells constantly interact with the surrounding microenvironment. Increasing evidence has demonstrated that the tumour microenvironment (TME) plays a considerable role in the development of various tumours,^11^ including SKCM,^12^ and targeting the TME could complement traditional treatment and improve the therapeutic response and clinical outcome for this malignancy.^11^, ^13^, ^14^ Infiltrating stromal and immune cells form the major fraction of normal cells in tumour tissues, and not only perturb the tumour signal in molecular studies but also play a vital role in cancer biology.^15^, ^16^ Previous studies have shown that melanoma cells deeply interact with the TME and the immune system^17^, ^18^ This knowledge has led to the identification of novel therapeutic targets and treatment strategies, including TME‐based predictive and prognostic biomarkers.^18^ Estimation of stromal and immune cells in malignant tumour tissues using the expression data (ESTIMATE) algorithm is an approach to study tumour‐infiltrating immune cells and their interactions with cancer cells to infer the fraction of immune and stromal cells in tumour samples underlying the expression of gene signatures.^16^, ^19^

## 2 MATERIALS AND METHODS

### 2.1 Data processing

The public RNA‐Seq data (fragments per kilobase of transcript per million fragments mapped) and mRNA expression profile data of SKCM from The Cancer Genome Atlas (TCGA, https://portal.gdc.cancer.gov) were retrospectively analysed, and relevant clinical data (sex, age, stage, tumour‐node‐metastasis (TNM) classification and survival data) were also obtained. A total of 471 cases of tumour tissue and one case of adjacent tissue were included. Moreover, 812 normal tissue samples were obtained from the Genotype‐Tissue Expression (GTEx) database.^20^, ^21^

### 2.2 TME scores generation

ESTIMATE is a tool to predict tumour purity, as well as the presence of infiltrating immune and stromal cells in tumour tissues.^16^ The ESTIMATE algorithm is based on single‐sample gene set enrichment analysis (ssGSEA) and mainly generates three scores: ImmuneScore (which infers the infiltration of immune cells in tumour tissues), StromalScore (which represents the presence of stromal cells in tumour tissues), and ESTIMATEScore (which captures tumour purity).^16^ Here, we generated StromalScore, ImmuneScore and ESTIMATEScore using the *ESTIMATE* package (version 1.0.13) to estimate the presence of immune and stromal cells in the TME for each tumour tissue. The higher the three TME scores, the larger the presence of the corresponding cells in TME.^22^ Tumour purity was negatively correlated with the three scores. Moreover, we introduced tumour purity as a supplement.

### 2.3 TME scores and clinicopathological characteristics

We generated four TME scores (ImmuneScore, StromalScore, ESTIMATEScore and tumour purity) and analysed the stage and TNM classification data of SKCM to determine the relationship between the ratio of stromal and immune components with clinicopathological characteristics.

### 2.4 Identification of differentially expressed prognostic genes

We grouped all SKCM samples into high or low score groups according to the median score of TME scores, and then applied the *survival* package (version 3.2‐7) and *survminer* package (version 0.4.8) to survival analysis.^23^ The Kaplan‐Meier method with the log rank test was used to plot the survival curve. We defined those that could group the population into different survival statistically as differential TME scores. After the identification of differential TME scores, we used the *limma* package (version 3.12) to screen differentially expressed genes in differential TME scores. We then used a Venn diagram to display the overlapping genes between differential TME scores and used these genes as differentially expressed prognostic genes.

### 2.5 Univariate Cox regression analysis

We performed univariate Cox regression analysis on differentially expressed prognostic genes to assess their correlation with SKCM prognosis and used the *forestplot* package (version 1.10.1) for visualization.

### 2.6 Development of a prognostic prediction model

Least absolute shrinkage and selection operator (LASSO) is a regression‐based method that allows numerous covariates in the model and thus could regulate those covariates that might influence the overall regression.^24^, ^25^ Here, we further obtained more reliable prognostic genes by LASSO to prevent overfitting of highly correlated genes from further filtering. The Kaplan‐Meier method with a log rank test was used to plot the survival curve of a single filtered gene and their combination. We used the *survivalROC* package (version 1.0.3) and *pROC* package (version 1.17.0.1) to draw receiver operating characteristic (ROC) curves and quantified the area under the ROC curves (AUCs) to evaluate the diagnostic performance of these genes alone and in combination. To verify the clinical significance of the differentially expressed prognostic gene‐based risk model, we used univariate and multivariate Cox regression analyses to determine the clinical relevance of the model. We then explored the possibility of using these genes as prognostic factors to independently affect the survival prognosis of SKCM. Finally, a prognostic prediction model was constructed to predict the overall survival (OS).

### 2.7 Identification of a novel prognostic biomarker and functional enrichment analysis

Based on previously established AUCs, we identified a novel prognostic biomarker. The *limma* package was used to evaluate the expression of the novel prognostic biomarker in SKCM tissues and normal tissues. Moreover, we also conducted clinicopathological characteristics analysis.

CIBERSORT is an algorithm that is widely used to characterize the cell composition of complex tissues through biomarker expression.^26^ The LM22 signature, a special genetic marker that contains 547 genes, is usually used to distinguish 22 immune cell subtypes downloaded from CIBERSORT. In this study, the *CIBERSORT* package was used and the LM22 signature algorithm was used to calculate the infiltration abundance of 22 immune cells between the high and low novel prognostic biomarker expression groups in 195 SKCM samples, including different T cells, B cells, plasma cells, natural killer cells and different myeloid subgroups. Subsequently, we also analysed the correlation between the expression distribution of 22 infiltrating immune cells in the high and low novel prognostic biomarker expression groups and used the *corrplot* package (version 0.84) to draw the correlation heat map.

Moreover, we grouped all SKCM samples into high and low expression groups according to the median expression of the novel prognostic gene and used the *ssGSEA* package^27^ to perform enrichment analysis of the Gene Ontology, Kyoto Encyclopedia of Genes and Genomes, and MsigDB dataset (msigdb.v7.0.entrez.gmt). The *ggplot2* package (version 3.3.3) was used for visualization. The flow chart was shown in Figure S1.

## 3 RESULTS

### 3.1 TME scores and clinicopathological characteristics

We generated four TME scores (ImmuneScore, StromalScore, ESTIMATEScore and tumour purity) and analysed the stage and TNM classification data of SKCM to determine the relationship between different TME cells and clinicopathological characteristics. Unfortunately, the results were not statistically significant (Figure S2), but the overall trends were relatively consistent.

### 3.2 Survival analysis of TME scores

**FIGURE 1:**
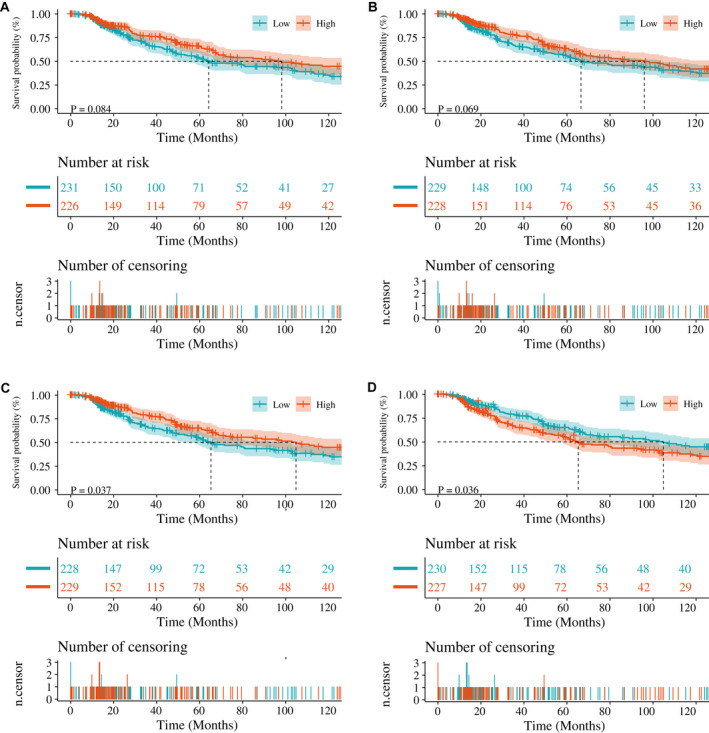
Correlation of TME scores with the survival of SKCM. A, ImmuneScore. B, StromalScore. C, ESTIMATEScore. D, Tumour purity. SKCM, Skin cutaneous melanoma; TME, tumour microenvironment

We analysed the correlation between the four TME scores and survival of SKCM patients. We found that there was no significant difference between StromalScore and SKCM OS (*p* = 0.084, Figure 1A) or ImmuneScore (*p* = 0.069, Figure 1B). In contrast, the ESTIMATEScore was positively correlated with OS (*p* = 0.037, Figure 1C), and tumour purity was negatively correlated with prognosis (*p* = 0.036, Figure 1D). Therefore, we considered the ESTIMATEScore and tumour purity as differential TME scores. Our results indicate that different TME cells might help predict the outcome of SKCM patients.

### 3.3 Screening of differentially expressed prognostic genes

**FIGURE 2:**
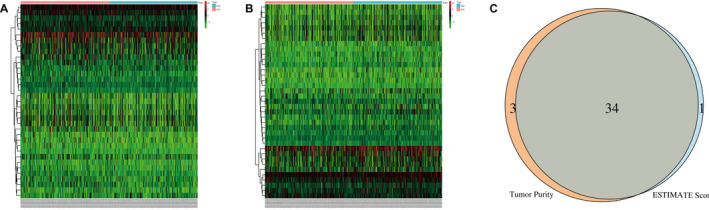
Screening of differentially expressed prognostic genes. A, Heat map of differentially expressed genes based on ESTIMATEScore. Red indicates genes that had higher expression level and blue indicates genes with lower expression in the different groups. B, Heat map of differentially expressed genes based on tumour purity. Red indicates genes that had higher expression level and blue indicates genes with lower expression in the different groups. C, Venn diagram analysis of differentially expressed genes based on ESTIMATEScore and tumour purity

We identified the differentially expressed genes in differential TME scores through a comparison analysis of high‐ and low‐differential TME score groups. There were 35 and 37 differentially expressed genes in ESTIMATEScore and tumour purity, respectively (Figure 2A,B). Using the intersection, we identified 34 differentially expressed prognostic genes (Figure 2C).

### 3.4 Univariate analysis

**FIGURE 3:**
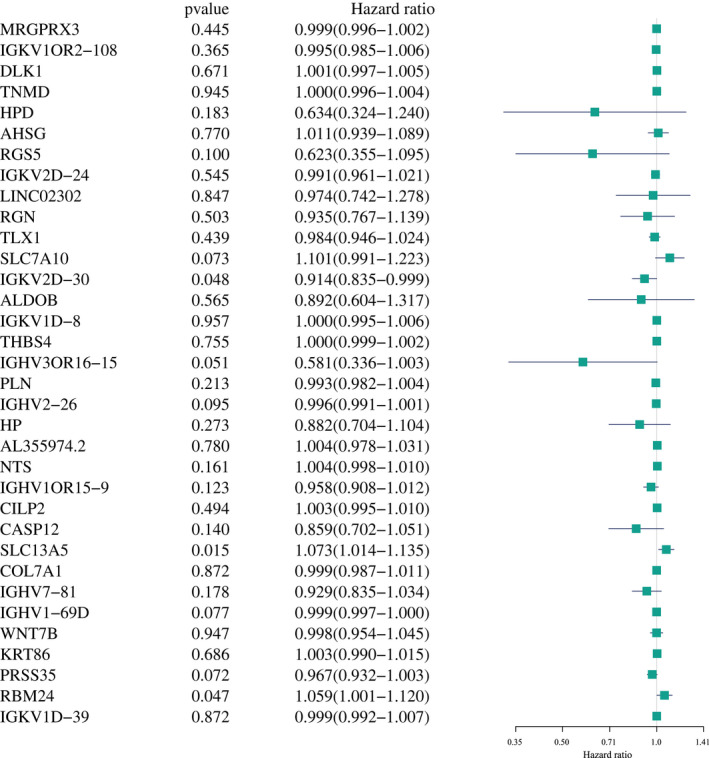
Univariate Cox regression analysis on differentially expressed prognostic genes

Univariate analysis revealed that the prognosis was correlated with SLC7A10 (*p* = 0.073), IGKV2D‐30 (*p* = 0.048), IGHV3OR16‐15 (*p* = 0.051), IGHV2‐26 (*p* = 0.095), SLC13A5 (*p* = 0.015), IGHV1‐69D (*p* = 0.077), PRSS35 (*p* = 0.072) and RBM24 (*p* = 0.047) (Figure 3).

### 3.5 Development of a prognostic prediction model

**FIGURE 4:**
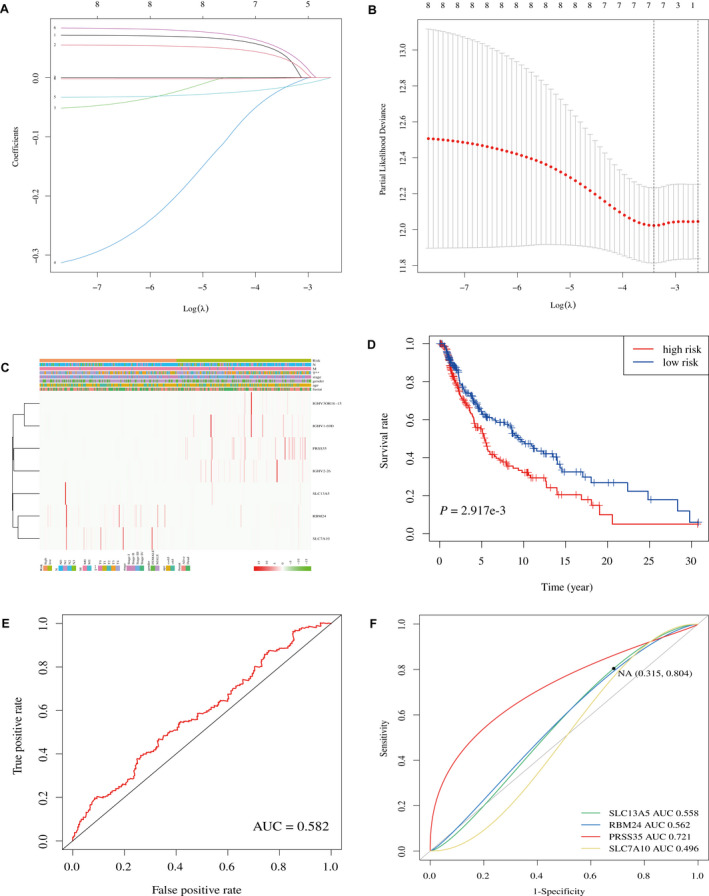
Development of prognostic prediction model. A, 10‐fold cross‐validation for turning parameter selection in the LASSO model. B, LASSO coefficient profiles of the seven differentially expressed prognostic genes. C, Heat map of clinical relevance. D, Survival curve of four differentially expressed prognostic genes. E, ROC of the combination of four differentially expressed prognostic genes. F, ROCs of SLC13A5, RBM24, PRSS35 and SLC7A10. LASSO, Least absolute shrinkage and selection operator; ROC, receiver operating characteristic

LASSO was used to generate the key differentially expressed prognostic genes. In LASSO, as logλ changed, the corresponding coefficients of certain differentially expressed prognostic genes were reduced to zero, indicating that their effects on the model could be omitted because they were shrinking parameters (Figure 4A). Following cross‐validation, a total of seven differentially expressed prognostic genes (SLC13A5, RBM24, IGHV3OR16‐15, PRSS35, SLC7A10, IGHV1‐69D and IGHV2‐26) achieved the minimum partial likelihood deviance (Figure 4B) and thus were used to develop the risk model. The clinical relevance heat map of the risk model showed the influence of the model on the clinical variables related to SKCM (Figure 4C). As IGHV3OR16‐15, IGHV1‐69D and IGHV2‐26 are pseudogenes, we only included SLC13A5, RBM24, PRSS35 and SLC7A10 in further analyses. The survival curves showed that whether the four differentially expressed prognostic genes were separated or combined together, they were able to effectively predict the outcome of SKCM patients (Figure 4D, Figure S3). The combination of four differentially expressed prognostic genes had a favourable diagnostic performance (AUC = 0.582, Figure 4E). Furthermore, we calculated the AUCs of each differentially expressed prognostic biomarker and concluded that PRSS35 had the best diagnostic performance (AUC = 0.721, Figure 4F); therefore, PRSS35 was considered as a novel prognostic biomarker.

**FIGURE 5:**
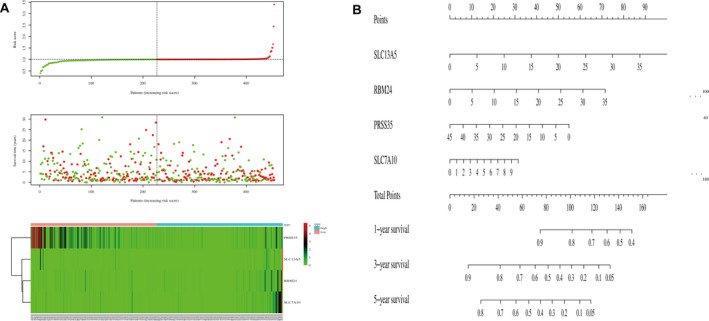
Characteristics of the prognostic prediction model and nomogram. A, Characteristics of the prognostic prediction model (top: the risk score of each SKCM patient; middle: overall survival and survival status of patients; bottom: heat map of gene expression profiles of SKCM patients). B, Nomogram. SKCM, Skin cutaneous melanoma

We compiled data from 346 SKCM patients with complete clinical information to perform univariate and Cox multivariate regression analyses. In the univariate analysis, several factors, such as age, stage, TNM classification and the risk score obtained by the constructed risk model, can affect the prognosis of SKCM (Table 1). In the multivariate Cox regression analysis, age, T classification, N classification and risk score could still affect the prognosis of SKCM (Table 1). This shows that the risk model we constructed can be utilized independent of other clinical traits and can aid in identifying prognostic biomarkers. Therefore, we determined the characteristics of the prognostic prediction model in SKCM patients (Figure 5A) and conducted a nomogram to predict the 1‐, 3‐ and 5‐year survival of SKCM patients (Figure 5B).

**TABLE 1:** Univariate and multivariate Cox regression analysis of prognostic prediction model

Characters	Univariate analysis		Multivariate analysis	
	HR (95%CI)	*p*‐Value	HR (95%CI)	*p*‐Value
Age	1.0195 (1.0085–1.0307)	**0.0005**	1.0119 (1.0008–1.0232)	**0.0357**
Gender	1.0319 (0.7326–1.4535)	0.8574	1.0429 (0.7369–1.4759)	0.8128
Stage	1.5671 (1.2851–1.9111)	**0.0000**	0.8556 (0.6052–1.2097)	0.3775
T	1.4445 (1.2363–1.6877)	**0.0000**	1.4973 (1.247–1.7978)	**0.0000**
M	2.6154 (1.0609–6.4475)	**0.0368**	2.2022 (0.8069–6.0105)	0.1233
N	1.4984 (1.2785–1.7562)	**0.0000**	1.632 (1.2861–2.0708)	**0.0001**
Risk score	3.6269 (2.0117–6.5387)	**0.0000**	3.5256 (1.7975–6.9153)	**0.0002**

Bold indicates *p* < .05.

### 3.6 Identification of a novel prognostic biomarker and functional enrichment analysis

**FIGURE 6:**
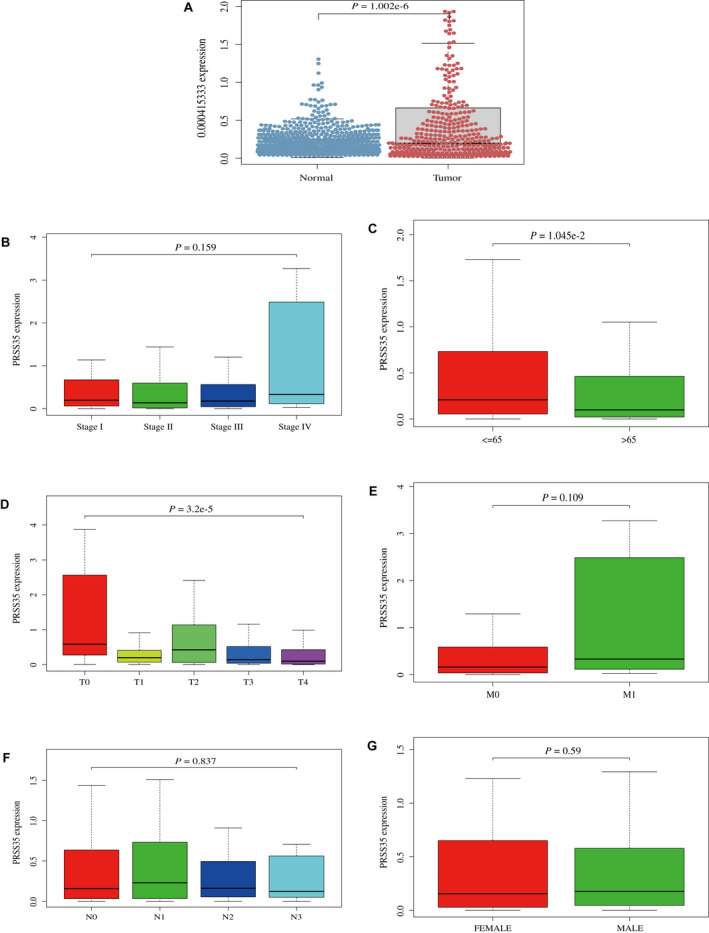
Expression of PRSS35 and clinical clinicopathological characteristics. A, Differentiated expression of PRSS35 in SKCM tissues and normal tissues. B, Correlation of PRSS35 expression with stage. C, Correlation of PRSS35 expression with age. D, Correlation of PRSS35 expression with T classification. E, Correlation of PRSS35 expression with M classification. F, Correlation of PRSS35 expression with N classification. G, Correlation of PRSS35 expression with gender

After identification of PRSS35 as a novel prognostic gene, we validated that PRSS35 was highly expressed in SKCM tissues compared to normal tissues (*p* < 0.0001, Figure 6A). Moreover, the exploration of clinical clinicopathological characteristics indicated that PRSS35 was associated with age (*p* = 0.0105) and T classification (*p* < 0.0001) (Figure 6B‐G), which was consistent with the results of the multivariate Cox regression analysis.

**FIGURE 7:**
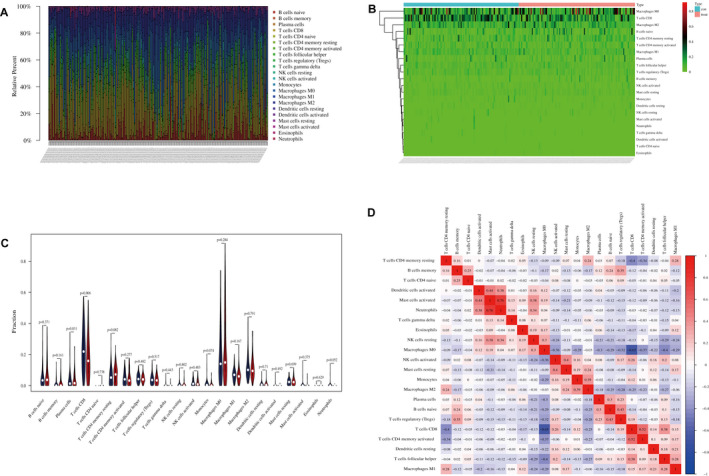
Immune infiltration analysis. A, Infiltration abundance. B, Heat map. C, Histogram. D, Correlation heat map. Red represents positive correlation, blue represents negative correlation, the darker the colour, the value closer to 1, the stronger the correlation

The infiltration abundance of 22 immune cells between the high and low PRSS35 expression groups in 195 SKCM samples is shown in Figure 7A,B. By further analysing 22 types of infiltrating immune cells, we found that CD8^+^ T cells were significantly high in tissues with low PRSS35 expression (Figure 7C). Subsequently, the correlation heat map showed that neutrophils and activated mast cells were positively correlated (*r* = 0.76), and CD8^+^ T cells were negatively correlated with M0 macrophages (*r* = −0.65) (Figure 7D). Combined with the above enrichment analysis results, the occurrence and development of SKCM may be related to inflammation and metabolic pathways, and may improve the distribution of immune cells by predicting the target small‐molecule drugs of CD8^+^ T cells, thereby improving the prognosis of SKCM.

**FIGURE 8:**
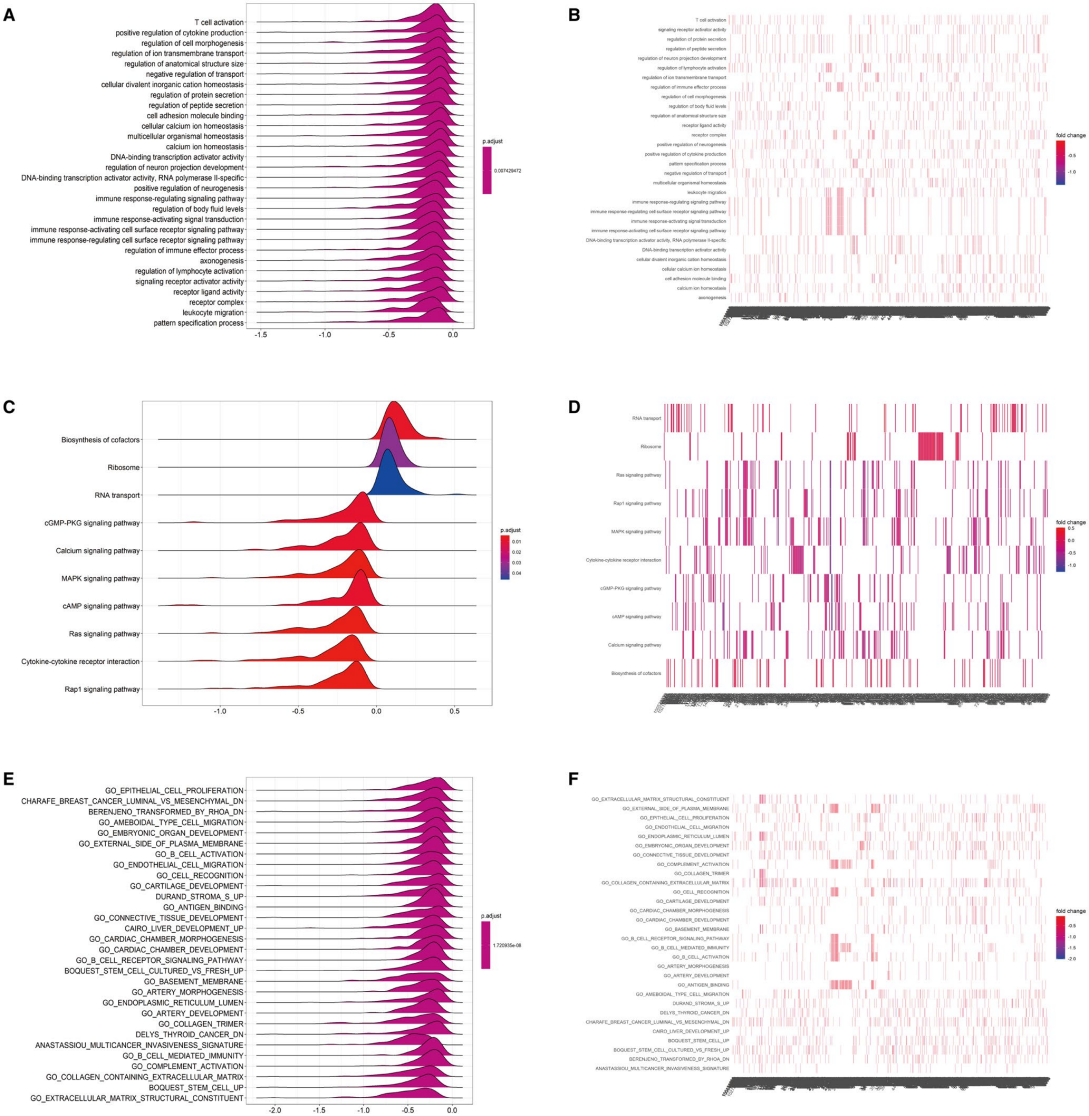
Functional enrichment analysis

The results suggest that it is functionally enriched in many immune‐related functions and important signalling pathways, such as in the regulation of lymphocyte activation, the immune response‐regulating cell surface receptor signalling pathway, and the immune response‐regulating signalling pathway, and is enriched in the MAPK signalling pathway, the interaction pathway between cell factors and cytokine receptors, as well as other signalling pathways, such as Rapl, Ras, cAMP and RNA transport (Figure 8). It is suggested that PRSS35 may regulate the TME and may affect the occurrence and development of SKCM by participating in the above enriched signalling pathways or by expressing immune‐related functions.

## 4 DISCUSSION

Skin cutaneous melanoma is one of the most aggressive malignancies worldwide. Despite advanced therapeutic methods, there are still persistent limitations, such as unsatisfactory sustained response, drug tolerance or resistance, toxicity and the considerable expense of these methods.^7^, ^28^ Moreover, there is still a lack of robust prognostic biomarkers to guide clinical decision‐making. Consequently, we attempted to identify a novel prognostic biomarker based on TME to contribute to SKCM therapy. In this study, we sought to develop a TME‐related prognostic biomarker and to classify TNM in SKCM patients through a combined analysis of multiple databases. We identified that PRSS35 is involved in the TME of SKCM. More importantly, we further concluded that PRSS35 might reflect the TME status of SKCM and act as a novel TME‐based prognostic biomarker in SKCM.

Tumour microenvironment has a considerable role in tumorigenesis and development. Transcriptome analysis of SKCM from TCGA showed that different immune components from the TME facilitate the prognosis of SKCM patients. Our results highlight the significance of exploring the interaction between SKCM cells and TME. The National Comprehensive Cancer Network recommends anti‐programmed cell death protein 1 monotherapy (pembrolizumab, nivolumab) as first‐line therapy for metastatic or unresectable SKCM and combination targeted therapy (dabrafenib/trametinib, vemurafenib/cobimetinib, encorafenib/binimetinib) as first‐line therapy for the BRAF V600‐activating mutation SKCM.^29^ Despite the promising efficacy of immune checkpoint inhibitors, approximately half of the patients either still have no durable response or suffer from immune‐related adverse events.^6^, ^7^, ^8^, ^9^, ^10^ Therefore, the universality of these regimens and the identification of patients who are more likely to have survival benefits from these treatments are unknown, and it is necessary to explore TME‐based prognostic biomarkers in SKCM. In our study, we started with the transcriptome analysis of SKCM from TCGA and found that the ESTIMATEScore and tumour purity were correlated with survival. We then identified seven potential prognostic markers and narrowed it down to PRSS35, which showed the most promise as a novel prognostic biomarker.

Serine proteases play an important role in cancer progression and metastasis.^30^, ^31^ As a member of the serine protease family, PRSS35 may also contribute to the aetiology of several cancers. Our results suggest that PRSS35 is upregulated in SKCM tissues, which is consistent with the results obtained from studies on ovarian cancer.^32^ Unfortunately, PRSS35 has not been reported to be immune‐related, except in aortic stenosis and aortic insufficiency.^33^ In other words, we report for the first time that PRSS35 upregulation is significantly associated with SKCM prognosis.

Furthermore, we uncovered the relationship between PRSS35 and TME. CIBERSORT indicated that immune‐related cells, such as CD8^+^ T cells, neutrophils, activated mast cells and M0 macrophages. It is well known that these are immune‐related cells.^34^ The GSEA results suggested that it was functionally enriched in many immune‐related functions and important signalling pathways, such as regulating lymphocyte activation, the immune response‐regulating cell surface receptor signalling pathway, immune response‐regulating signalling pathway, and is enriched in MAPK signalling pathways, the interaction pathways between cell factors and cytokine receptors, as well as signalling pathways, such as Rapl, Ras, cAMP and RNA transport. These enriched functions and signalling pathways indicated that PRSS35 might reflect the TME status of SKCM.

There are some limitations to this study, although the established novel TME‐based prognostic biomarker in SKCM is powerful. First, the transcriptome data used in the present study were obtained from TCGA and GTEx databases. Although we conducted homogenization, the source bias could not be ignored and might have affected the extrapolation of our results. Second, we were not able to obtain satisfactory results in the relationship between TME scores/PRSS35 and clinicopathological characteristics due to the limited clinical information and clinical sample size included in our study. Third, only one SKCM data set was applied in the present study; however, more data sets with richer clinical information and a larger sample size were collected to verify our findings. Finally, this was an *in silico* analysis; therefore, molecular biology experiments, as well as prospective, well‐designed, multicentre studies are required to validate the findings.

In conclusion, we obtained TME scores using ESTIMATE and screened the differentially expressed prognostic genes after the identification of differential TME scores, and finally identified PRSS35 as a novel prognostic biomarker. LASSO, Cox regression and functional enrichment analyses were also conducted using CIBERSORT and ssGSEA. By investigating the TME to obtain new insights for the clinical diagnosis and treatment of SKCM, our study determined that PRSS35 might act as a potential prognostic biomarker for this malignancy.

## CONFLICT OF INTEREST

The authors have no conflict of interest to declare.

## AUTHOR CONTRIBUTIONS

**Rong‐Hua Yang:** Data curation (equal); Investigation (equal); Software (equal); Validation (equal); Visualization (equal). **Bo Liang:** Data curation (equal); Investigation (equal); Software (equal); Validation (equal); Visualization (equal); Writing‐original draft (lead). **Jie‐Hua Li:** Data curation (equal); Formal analysis (equal); Methodology (equal). **Xiao‐Bing Pi:** Data curation (equal); Resources (equal); Validation (equal). **Kai Yu:** Data curation (equal); Resources (equal); Validation (equal). **Shi‐Jian Xiang:** Formal analysis (equal); Resources (equal); Software (equal). **Ning Gu:** Investigation (equal); Methodology (equal); Resources (equal); Writing‐original draft (supporting). **Xiao‐Dong Chen:** Funding acquisition (equal); Project administration (equal); Supervision (equal); Writing‐review & editing (equal). **Si‐Tong Zhou:** Funding acquisition (equal); Methodology (equal); Project administration (equal); Supervision (equal); Writing‐review & editing (equal).

## Supporting information

Fig S1

Fig S2

Fig S3

## Data Availability

Data sharing is not applicable to this article as no new data were created or analysed in this study.
