# Supplementary figures and images for: Identification of a novel tumour microenvironment‐based prognostic biomarker in skin cutaneous melanoma
Source: J Cell Mol Med. 2021 Nov 10;25(23):10990–1001. doi: 10.1111/jcmm.17021 (PMC8642691; doi:10.1111/jcmm.17021)

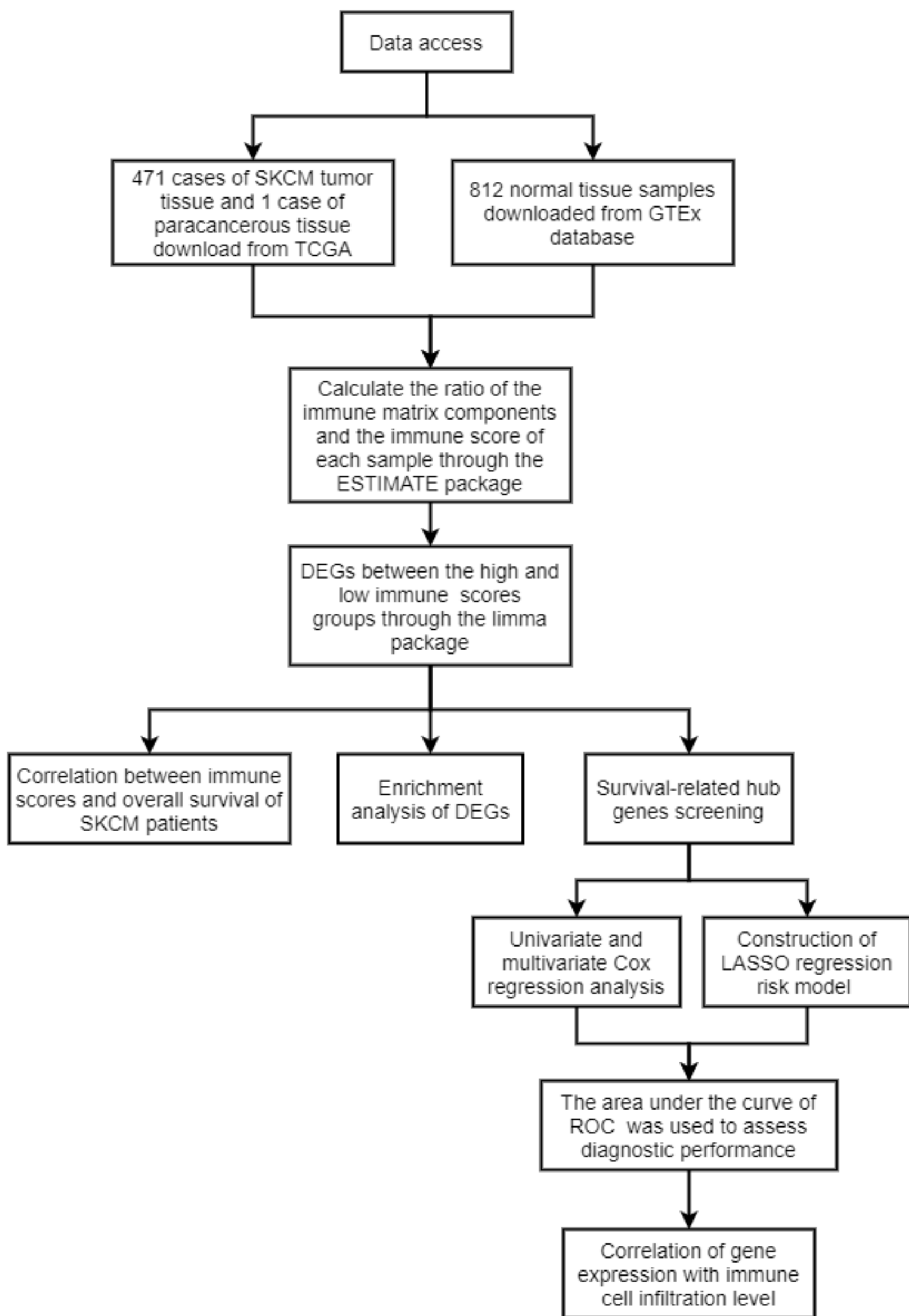

Supplement: Supplementary file 1 — Fig S1 [file JCMM-25-10990-s002.pdf]

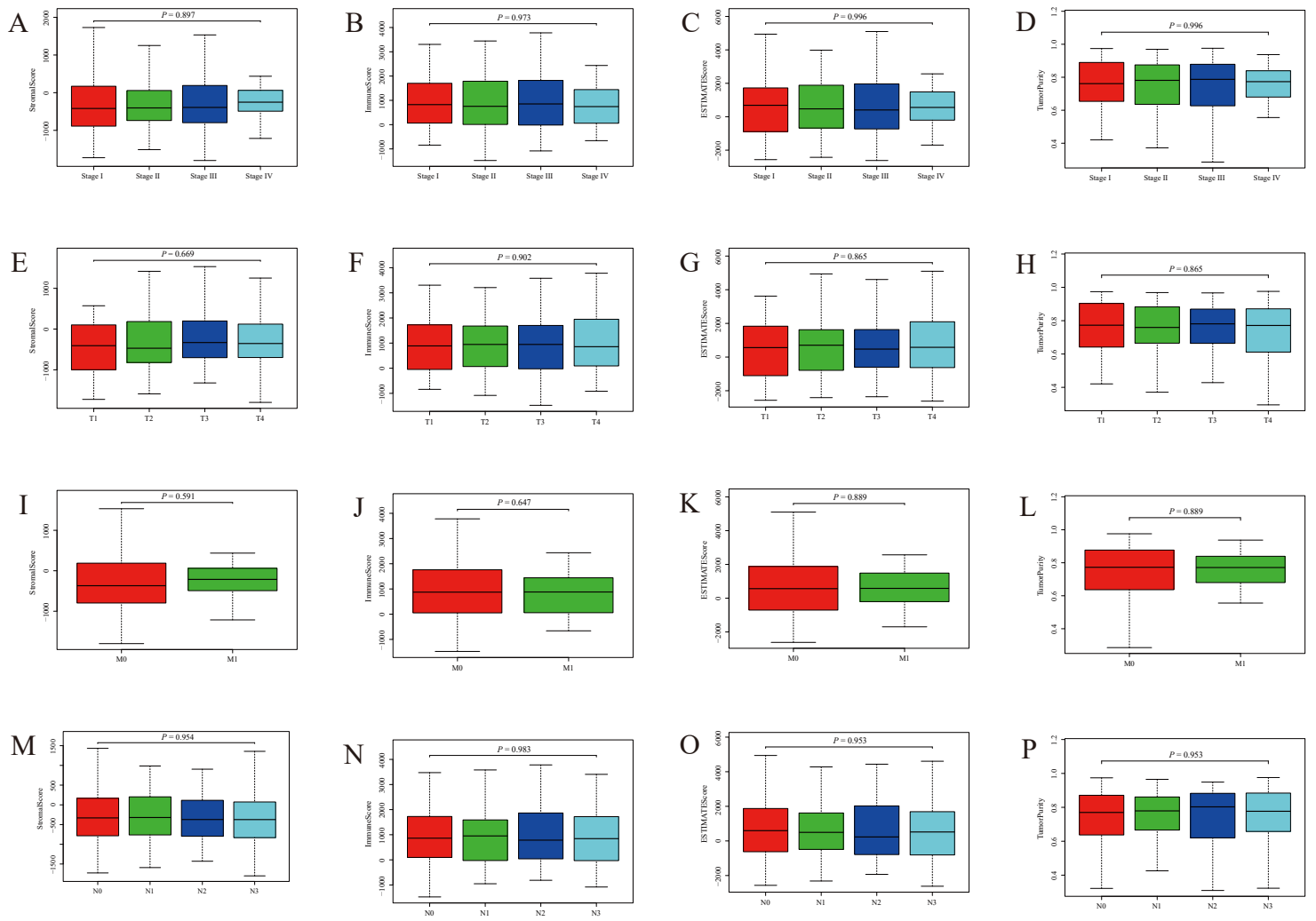

Figure S2

Supplement: Supplementary file 2 — Fig S2 [file JCMM-25-10990-s003.pdf]

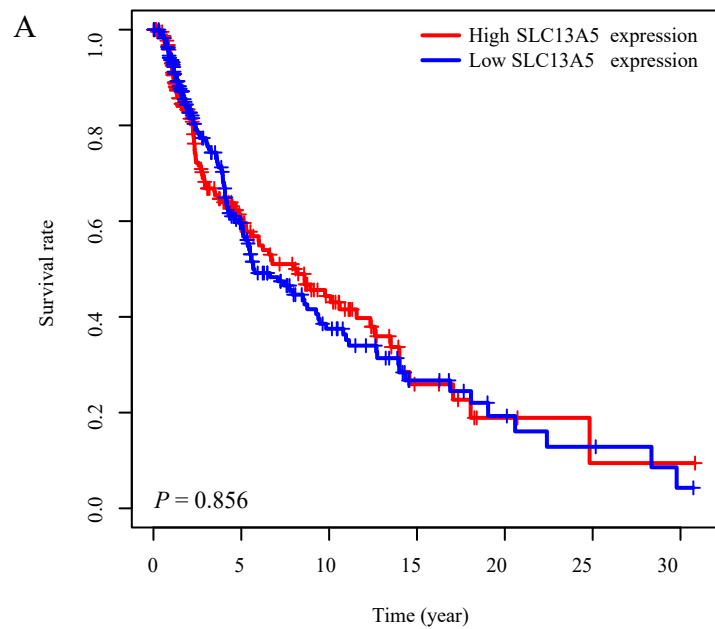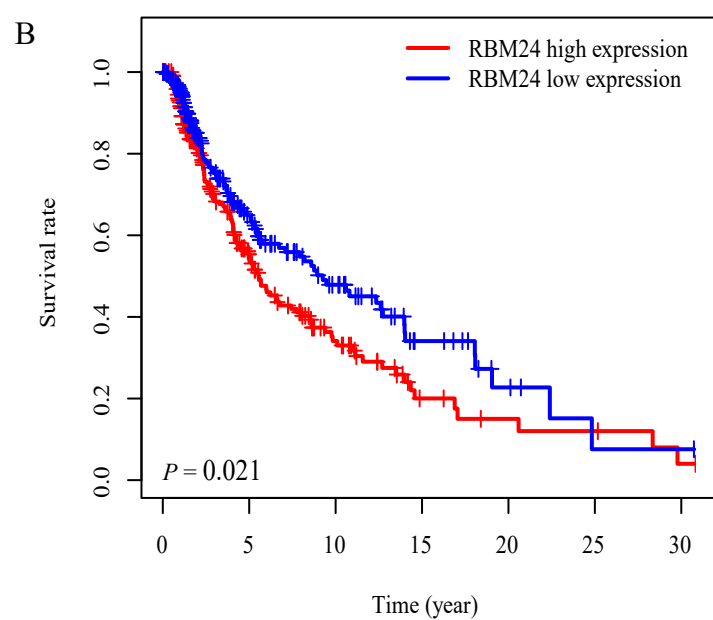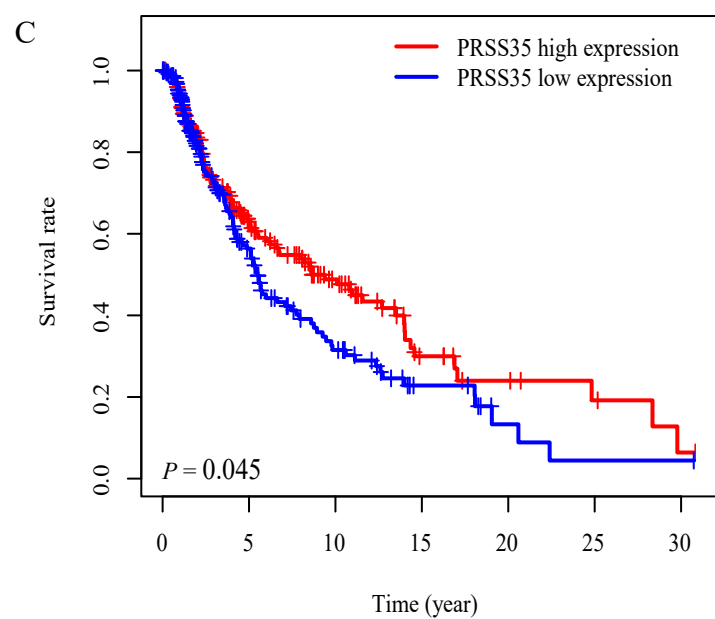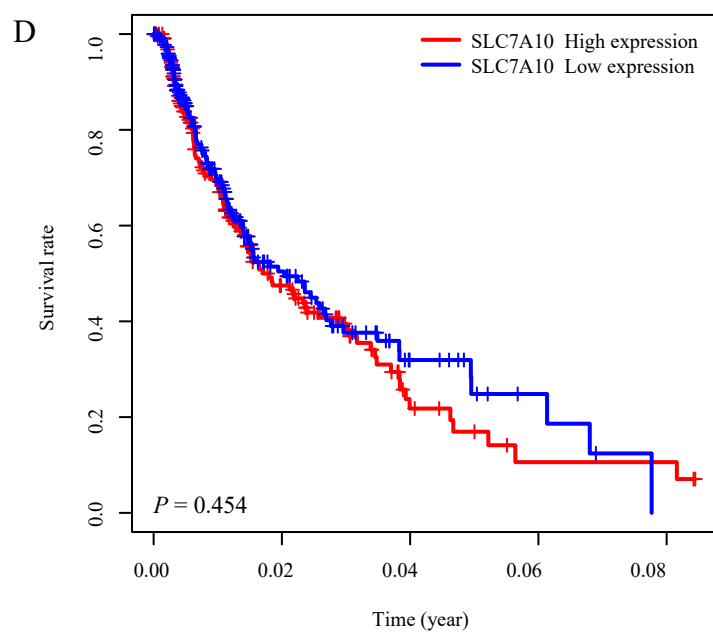

Figure S3

Supplement: Supplementary file 3 — Fig S3 [file JCMM-25-10990-s001.pdf]
